# Cognitive and Socio-Emotional Deficits in Platelet-Derived Growth Factor Receptor-β Gene Knockout Mice

**DOI:** 10.1371/journal.pone.0018004

**Published:** 2011-03-18

**Authors:** Phuong Thi Hong Nguyen, Tomoya Nakamura, Etsuro Hori, Susumu Urakawa, Teruko Uwano, Juanjuan Zhao, Ruixi Li, Nguyen Duy Bac, Takeru Hamashima, Yoko Ishii, Takako Matsushima, Taketoshi Ono, Masakiyo Sasahara, Hisao Nishijo

**Affiliations:** 1 System Emotional Science, Graduate School of Medicine and Pharmaceutical Sciences, University of Toyama, Toyama, Japan; 2 Department of Judo Physiotherapy, Graduate School of Medicine and Pharmaceutical Sciences, University of Toyama, Toyama, Japan; 3 Integrative Neuroscience, Graduate School of Medicine and Pharmaceutical Sciences, University of Toyama, Toyama, Japan; 4 Department of Pathology, Graduate School of Medicine and Pharmaceutical Sciences, University of Toyama, Toyama, Japan; Tokyo Medical and Dental University, Japan

## Abstract

Platelet-derived growth factor (PDGF) is a potent mitogen. Extensive *in vivo* studies of PDGF and its receptor (PDGFR) genes have reported that PDGF plays an important role in embryogenesis and development of the central nervous system (CNS). Furthermore, PDGF and the β subunit of the PDGF receptor (PDGFR-β) have been reported to be associated with schizophrenia and autism. However, no study has reported on the effects of *PDGF* deletion on mice behavior. Here we generated novel mutant mice (PDGFR-β KO) in which *PDGFR-β* was conditionally deleted in CNS neurons using the Cre/loxP system. Mice without the *Cre* transgene but with floxed *PDGFR-β* were used as controls. Both groups of mice reached adulthood without any apparent anatomical defects. These mice were further examined by conducting several behavioral tests for spatial memory, social interaction, conditioning, prepulse inhibition, and forced swimming. The test results indicated that the PDGFR-β KO mice show deficits in all of these areas. Furthermore, an immunohistochemical study of the PDGFR-β KO mice brain indicated that the number of parvalbumin (calcium-binding protein)-positive (i.e., putatively γ-aminobutyric acid-ergic) neurons was low in the amygdala, hippocampus, and medial prefrontal cortex. Neurophysiological studies indicated that sensory-evoked gamma oscillation was low in the PDGFR-β KO mice, consistent with the observed reduction in the number of parvalbumin-positive neurons. These results suggest that PDGFR-β plays an important role in cognitive and socioemotional functions, and that deficits in this receptor may partly underlie the cognitive and socioemotional deficits observed in schizophrenic and autistic patients.

## Introduction

The family of platelet-derived growth factors (PDGF) comprises 4 members—PDGF-A, -B, -C, and -D—that are assembled from disulfide-linked homo- or heterodimers of 2 distinct but related chains (PDGF-AA, -AB, -BB, -CC, and -DD). Two receptor subtypes of PDGF (PDGFR-α and -β) can form mature dimeric receptor complexes that can bind to ligands with different affinities [1]. PDGFR-α is largely expressed in oligodendroglial progenitors, while PDGFR-β is predominantly expressed in neurons [2] and upregulated in the neonatal rat brain [3]. PDGF-BB that specifically binds to PDGFR-ββ is abundantly expressed in neurons and is upregulated in neonatal brains [4]–[6].

Altering PDGFR-β may result in abnormalities during the development of the central nervous system (CNS) due to the loss of paracrine and autocrine stimulation [7], [8]. Consistent with this hypothesis, evidence of the relationship between alteration of PDGFR-β and -BB and human psychiatric disorders has been reported. According to linkage analyses, *PDGFR-β* is located on chromosome 5q31-q32 [9], which contains susceptibility genes for schizophrenia [10]–[17]. Following the first report by Sherrington et al. [18] that indicated the association between *PDGFR-β* and schizophrenia, several researchers demonstrated that *PDGFR-β* affects neurotransmitter systems related to schizophrenia and/or autism. D2 dopamine receptor-mediated transactivation of PDGFR-β alters excitatory neurotransmission mediated by the N-methyl-D-aspartate subtype of glutamate receptors [19]. PDGF exerts neurotrophic effects on both γ-aminobutyric acid (GABA)ergic and dopaminergic neurons [3], [20], [21] and has long-lasting effects on N-methyl-D-aspartate receptor-mediated synaptic transmission in the hippocampus [22], [23]. Furthermore, recent studies reported that 3 single nucleotide polymorphisms and 2 haplotypes of *PDGFR-β* are associated with schizophrenia [24], and that serum levels of PDGF-BB are high in autistic boys [25].

To investigate the role of *PDGFR-β* in CNS development in vivo, conditional knockout (KO) mice with suppressed expression of neuronal *PDGFR-β* in the Cre/loxP system (PDGFR-β KO mice) were generated [26]. However, no apparent anatomical defects were observed in these mice. The association of *PDGFR-β* and PDGF-BB with schizophrenia and autism in humans (see above) strongly suggests that the PDGFR-β KO mice show cognitive and socioemotional deficits. Here the PDGFR-β KO mice were examined using a battery of behavioral tests designed to analyze such deficits. Furthermore, parvalbumin-positive neurons, which are mostly GABAergic and closely involved in the pathologies of schizophrenia and autism [27]–[29], were analyzed in the amygdala, hippocampus, and medial prefrontal cortex. Evoked gamma oscillation associated with GABAergic neurons, which was reduced in schizophrenia [30]–[32], was also analyzed.

## Methods

### Ethics statement

All mice were housed in individual cages in a temperature-controlled environment with a 12/12-h light/dark cycle (lights were turned on and off at 08:00, and 20:00, respectively). Food and water was supplied ad libitum. Mice (10- to 16-week-old) were handled for 3 consecutive days before the start of the experiments. All experimental protocols were performed in accordance with the guidelines for care and use of laboratory animals approved by the University of Toyama and the National Institutes of Health's *Guide for the Care and Use of Laboratory Animals*, and approved by the Committee for Animal Experiments at the University of Toyama. (License number: S-2009MED-9).

### Generation of conditional PDGFR-β KO mice

The Cre/loxP system was used to develop conditional PDGFR-β KO mutants. A previously established mutant mouse line was used, in which exons 4–7 of *PDGFR-β*, which encode the extracellular domain of the PDGFR-β protein, were flanked by 2 loxP sequences (floxed) positioned in introns 3 and 7 [33]. After *Cre*-mediated recombination, deletion of the loxP-flanking region and resulting frame shift mutation in the adjoining 3′ region occurred in *PDGFR-β*. To obtain conditional PDGFR-β KO, we then crossed mutant mice harboring the *PDGFR-β* floxed allele and those expressing *Cre* recombinase under the control of the nestin promoter and enhancer (*nestin-Cre* ^+^ mouse, The Jackson Laboratory, Bar Harbor, ME, USA) as previously described [26], [34]. Before this cross, both mutant mice harboring floxed *PDGFR-β* and *nestin-Cre^+^* were outbred to the mice of C57BL/6J (B6/J) strain for 14 generations to replace the genetic background of our mutant mice with that of the B6/J strain. In the present study, the following 2 types of 10- to 16-week-old male mice were used: mice with the *Cre* transgene and floxed *PDGFR-β* (PDGFR-β KO mice) and mice without the *Cre* transgene but with floxed *PDGFR-β* (control mice).

Genotypes were confirmed by PCR of tail DNA, using oligonucleotide primers pairs for floxed *PDGFR-β* and for the *Cre* transgene as described previously [26]. The genotyping was confirmed by Western blot of the total lysates of the adult mouse brains to show that the PDGFR-*β* expression decreased to undetectable levels in the PDGFR-β KO mice compared with that in the control mice [26]. A total of 41 control male mice born from 10 dams and 41 PDGFR-β KO male mice born from 21 dams were used in the present study. The following all behavioral, histological, and neurophysiological testing was conducted by experimenters blind to genotype.

### Hot plate test

Sensitivity to thermal nociception [35] was evaluated using a commercially available hot plate analgesia meter (Model DS37, Ugo Basile, UK). The apparatus consisted of a metal plate (24.5×24.5 cm), which could be heated to a constant temperature, on which a plastic cylinder (20 × 18 cm; diameter × height) was placed. Mice were brought to the testing room and allowed to acclimate for 10 min prior to the test.

The latency to respond to the thermal stimulus (56.0±0.1°C) was defined as the time between the moment the mouse was placed inside the cylinder and when it licked or flicked its hind paws or jolted or jumped off the hot plate. Each animal was tested once per session.

### Food search test

Mice were individually placed into the apparatus used for the food search test (KUROBOX, Phenotype Analyzing Co., Ltd., Nagasaki, Japan) [36]. The apparatus consisted of 2 compartments. The nest compartment (80×130×210 mm; length × width × height) was separated from the observational compartment (240×240×210 mm) by a partition, and the mouse could freely move through the partition into the square observation field. Barycentric coordinates of each mouse were recorded using 64 infrared photosensors.

The nest compartment was covered to block light for 24 h. The observation compartment was maintained in the 12/12-h light/dark cycle. Food stations were set up at the 4 corners of the observational compartment. Each food station consisted of a polyvinylchloride wall unit (30×45×20 mm) and a food cup containing powdered food. Although all 4 food cups were filled with the powdered food, a mesh shutter covered 3 of the 4 food cups. The mice did not have access to the food in the portions covered by the mesh shutter; however, the same olfactory stimulus emanated from all food cups. The rotary feeder moved the meshed shutter in a counter-clockwise direction so that the food station changed every 4 h. At any given time, the mice could only take food through a single station that was not covered by the mesh shutter. The supply of water was ad libitum in the observation compartment.

The barycentric coordinates of each mouse were recorded every second. Regions of interest (ROIs) were established at all 4 corners of the observational field. Each ROI consisted of a 60×60 mm^2^ area. When the barycenter of a mouse remained within the same ROI for more than 4 s, this was counted as a visit to that food station. The locomotion of each mouse over a period of 4 days, number of visits to each ROI, and the order of these visits, which were numbered until the mouse returned to the nest, were recorded. Correct visit ratio was defined as the ratio of the number of visits to the correct food station to the number of visits to all stations.

### Social interaction test

The testing cage consisted of a white plastic box (38.5×22.5×20 cm). Individual mice were allowed to acclimate to the testing cage for 20 min prior to the test. Pairs of mice from the same group were placed in opposite corners of the box. Their activities in the box were recorded using an overhead CCD camera for 30 min. Frequencies and durations of social activities (e.g., proximity behavior, approaching and leaving, following, social sniffing, active and passive contact, and mounting) were automatically analyzed using the SocialScan program (CleverSys Inc., Reston, VA, USA). The plastic box was wiped with 70% ethanol and air dried between the trials.

Social contact was defined as interbody distances between 2 mice of less than 20 mm. Furthermore, social contact was divided into the following 2 subcategories: active and passive social contact. When 1 mouse approached and actively contacted another mouse, the mouse's behavior was considered active and the other mouse's behavior was considered passive. These 2 types of social contact were defined as follows: the active approaching mouse to move faster than 40 mm/s within 15 frames (corresponding to 0.5 s) and the ratio of the “movement” of the active mouse to that of the passive mouse to be greater than 2.0. Here, “movement” is defined as M(n) = 1 – Intersect(A(n), A(n+1))/Union(A(n), A(n+1)), where A(n) is denoted as the animal area at previous frame n.

Approaching was defined by the direction, speed, and distance covered. The direction criteria required the approaching mouse to move towards the other mouse, and the angle between the direction of the approaching mouse, which was defined as the head direction detected by the mouse nose, and the line to the other mouse to be less than 45°. The speed criteria required the approaching mouse to move faster than 30 mm/s and the other mouse to move slower than 100 mm/s within 15 frames (0.5 s). The distance criteria required the approaching mouse to travel at least 30 mm and the distance between the 2 mice to be less than 1000 mm. The duration of each approaching was defined as the continuous duration of such action that lasted at least for 2.0 sec. At the beginning of the approach, two animals should not be very close, by default, inter distance more than 100 mm. At the ending of the approach, two animals should be quite close.

Social sniffing was defined as the distance between the nose of the sniffing mouse and the body of the other mouse being sniffed of less than 30 mm. The duration of each social sniffing was defined as the continuous duration of such action that lasted at least for 0.27 sec.

Social leaving was also defined by the direction, speed, and distance covered. The direction criteria required the leaving mouse to leave the other mouse first, and the angle between the direction of the leaving mouse and the line from the leaving mouse to the other mouse to be greater than 100°. The speed criteria required the speed of the leaving mouse to be greater than 30 mm/s within 15 frames (0.5 s). The distance criteria required the distance between the 2 mice to be less than 1000 mm, and the leaving mouse to travel at least 30 mm. Continuous actions meeting these criteria and lasting for at least 0.5 s were considered social leaving.

Social following was defined by the following criteria: (1) the angle between the direction of the leaving mouse and the direction of the following mouse to be less than 90°, (2) the angle between the direction of the following mouse and the line from the following mouse to the leaving mouse to be less than 30°, (3) the angle between the direction of the leaving mouse and the line from the leaving mouse to the following mouse to be greater than 100°, (4) the distance between the 2 mice to be less than 300 mm, (5) both the following and leaving mice to move at least 5 mm within 15 frames (0.5 s), and (6) the following mouse to travel at least 30 mm within 15 frames (0.5 s). Continuous actions meeting these criteria and lasting for at least 0.5 s were considered social following.

Social mounting was defined as a contact in which two-thirds of the head or body of 1 mouse rode on top of the other mouse for at least 20 frames (0.6 s). It is detected by the joint shape change of two animals.

### Contextual and cued fear conditioning

On day 0, each mouse was placed in a testing chamber (11×11×12.5 cm; O'hara & Co, Ltd., Tokyo, Japan) inside a sound-attenuated chamber with a brightness of 200 lux and background white noise of 50 dB. Each mouse was allowed to explore freely for 5 min. The chamber was equipped with a barred metal floor that could deliver an electric shock. Initially, the baseline level of freezing behavior was recorded for 5 min. Then, a 10 kHz, 65-dB tone, which served as the conditioned stimulus, was presented for 20 s. This tone was followed by a footshock (0.4 mA for 1 s), which served as the unconditioned stimulus. Four more conditioned stimulus/unconditioned stimulus pairings were presented with an interstimulus interval of 465–870 s. Freezing behavior was analyzed for 20 s after the presentation of each conditioned tone.

A contextual test was conducted 24 (day 1), 48 (day 2), and 72 h (day 3) after conditioning in the same chamber without the presentation of the tone or footshock. The mice were left in the chamber for 11 min, and the freezing time during the initial 5 min was analyzed. The animals were then returned to their home cages. A cued test was conducted in a different environment 2 h after the contextual test using a white plastic box with a brightness of 50 lux, background white noise of 60 dB, and presence of the smell of alcohol. The same conditioned tones were presented 5 times on each experimental day without the footshock after the baseline level of freezing behavior was assessed for 5 min. Freezing behavior was analyzed for 20 s after the presentation of each tone.

Freezing behavior was analyzed using video-captured images of the mice. Images of the mice were captured at the rate of 1 frame/s. For each pair of successive frames, the distance (in pixels) covered by the mouse was measured. When this distance was below a certain threshold (20 pixels), the behavior was judged as “freezing.” This optimal threshold (number of pixels) to judge freezing was determined by adjusting it to the amount of freezing measured by human observation.

### Prepulse inhibition (PPI)

The startle reflex measurement system (O'Hara & Co.) was used to measure PPI. A test session began by placing a mouse in a plastic cylinder in a sound-attenuated chamber and leaving it undisturbed for 10 min. The background white noise level in the chamber was 65 dB. The prepulse sound (0, 70, 72, 74, 78 and 82 dB) was presented for 120 ms before the startle stimulus [120 dB white noise (40 ms), main pulse] was provided. Each test session was composed of 36 trials. Six blocks of the 6 prepulse/main pulse combination types were presented in a pseudorandom order, such that each combination type was presented once within a block. The average intertrial interval was 15 s (range, 10–20 s). Startle responses were recorded for 140 ms (measuring the response every 1 ms), starting with the onset of the prepulse stimulus. These responses were corrected for the body weight of each mouse. The PPI percentage was calculated using the following formula: [(startle amplitude in trials without prepulse) − (startle amplitude in trials with prepulse)]/(startle amplitude in trials without prepulse) ×100.

### Forced swim test (FST)

FST used in this study was based on the original version used for mice by Porsolt with modifications. Mice were placed in a cylinder (15 cm×22.5 cm; diameter × height) filled with water (15 cm high). The mice were not able to touch the bottom. The water temperature was set at 25±1°C. The cylinder was placed in a box with infrared cell sensors on the walls to detect swimming activity (SCANET, Melquest Inc., Toyama, Japan). The software set up a rectangle that circumscribed the body of an animal every 0.3 sec. If the animal went out of the rectangle (i.e., a part of the animal body was detected out side the rectangle) 0.3 sec after setting up the rectangle, the software counted as “movement (swimming)” in that period. “Immobility” was defined as such if the animal stayed within the same rectangle 0.3 sec after setting up the rectangle.

On the first day, the mice were placed in water and forced to swim in a single trial of 15 min. On the second day, the mice were placed in water and forced to swim in a single trial of 5 min. Swimming time was continuously recorded every 1 min of each trial in each animal. After the test, the animals were dried with towels and returned to their cages.

### Immunohistochemistry

Under deep anesthesia with sodium pentobarbital (50 mg/kg body weight, i.p.), the mice were transcardially perfused with heparanized saline (0.9% w/v NaCl), followed by 4% paraformaldehyde dissolved in 0.1 M phosphate buffer (PB) (pH 7.4). After perfusion, the brains were removed from the skull, coronarily cut into small blocks, and postfixed in 4% paraformaldehyde overnight. The fixed brain blocks were immersed in 30% sucrose dissolved in 0.1 M PB until they sank down to the bottom. The brain blocks were then frozen in dry ice and coronarily cut into 40 µm-thick sections. The sections were placed in 0.01 M phosphate buffer saline (PBS) and then transferred into an antifreeze solution (25% ethylene glycol, 25% glycerin, and 50% 0.1 M PB) and stored at −20°C until immunohistochemical staining could be performed.

The sections were washed thrice in 0.01 M PBS for 15 min, blocked with 3% normal horse serum in PBS for 30 min at room temperature, and incubated overnight at 4°C with mouse monoclonal anti-parvalbumin antibodies (1∶10,000 dilution in 1% horse serum PBS, Sigma, St. Louis, MO, USA). These sections were washed thrice with 0.01 M PBS for 10 min each time, incubated with biotinylated horse anti-mouse IgG (1∶200 dilution, Vector, Burlingame, USA) for 50 min at room temperature, and then, after washing, incubated in ABC reagent (Vector) for 50 min. Finally, the parvalbumin-immunoreactive elements were visualized by reacting them with 25 mg 3,3′-diaminobenzidine and 30 µl 30% H_2_O_2_ in 100 ml 0.01 M PBS (pH 7.4) for 5–8 min. The sections were then rinsed several times in PBS, dehydrated in graded concentrations of ethanol, cleared in xylene, and cover-slipped with Entellan (MercK, Darmstadt, Germany). Negative control sections were treated identically except for omission of the primary antibody. No reaction product was observed in any of the control sections.

To count the number of parvalbumin-positive neurons, 3 sections of each level of the brain were selected from each animal, and digital images of the stained sections were captured at +1.1, +1.3, and +1.4 mm AP from bregma (medial prefrontal cortex, anterior cingulate cortex, infralimbic and prelimbic areas), −0.5, −0.7, −1.1, −1.6, and −2.1 mm AP (amygdala), and −1.6 and −2.1 mm AP (dorsal hippocampus). These digital images were analyzed using the ImageJ software (NIH ImageJ; http://rsbweb.nih.gov/ij/). Anatomical structures were delineated on the basis of the atlas of the C57BL/6 mouse brain provided by Hof et al. [37]. Cell counts in the sections above the AP level were averaged in each animal.

### Neurophysiological recordings of auditory event-related gamma oscillation

In this experiment, naive control (n = 10) and PDGFR-β KO (n = 9) were used (the animals received no other behavioral testings). Under anesthesia with avertin (187.5 mg/kg, i.p.), 2 screws, which later worked as EEG electrodes, were implanted over the frontal cortex and cerebellum of the skulls of the mice. A connector for the wires was connected to the screws and attached to the skull using cranioplastic. After recovery from surgery (1 week), the animals were acclimated to all of the handling and testing procedures described previously. On the days when measurements were recorded in the dark phase, the animals were put into a plastic recording box (235×185×125 mm). Signals from the electrodes were amplified and band-pass filtered at 1.5–1000 Hz (3 dB corner, 6 dB octave/slope) using an amplifier (MEG-5200G, Nihon Kohden, Tokyo, Japan). The amplified analog signals were digitized at 2 kHz and stored using the Micro 1401mkII hardware and Spike2 (ver. 6) software (Cambridge Electronic Design, Cambridge, UK). Auditory stimuli (5 kHz pure tone, 500 ms duration) were generated using a sound generator (DPS-725T, DIA Medical System, Tokyo, Japan) and delivered using a speaker after amplification to 85 dB at an interval of 3 s with a background noise of 50 dB.

Recent studies have reported a reduction in early phase-locked gamma oscillation (evoked gamma responses; ERPs) as a characteristic of schizophrenia [30]–[32]. In the present study, ERPs in response to auditory stimulation were analyzed [38]. ERPs recorded in individual trials were digitally band-pass filtered between 30–80 Hz using infinite impulse response filters and then averaged across the trial for each animal. Phase synchronized gamma oscillation with respect to stimulus onset would survive the averaging process and can be seen in the averaged ERPs. The averaged gamma data were rectified by squaring to produce a positive value for gamma activity at each point. This enabled the measurement of the area under the curve (AUC) for the time window between 0–100 ms from stimulus onset. The mean AUC across the animals was compared between the 2 groups of mice.

### Statistical data analysis

Quantitative data were expressed as the mean ± SEM. The data were analyzed by two-way mixed repeated measures ANOVA based on general linear model followed by Bonferroni test, or by Student's t-tests using SPSS 19.0 (SPSS Inc., Chicago, IL). The statistical significance level was set at *p*<0.05.

## Results

### Social interaction

**Figure 1:**
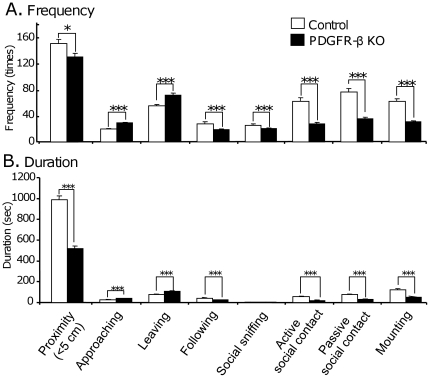
Social interactions of the PDGFR-β KO and control mice. Frequencies (A) and durations (B) of each social behavior are indicated. Black columns, PDGFR-β KO mice; white columns, control mice; *, *p*<0.05; **, *p*<0.01; and ***, *p*<0.001 (Student's t-test).

Figure 1 shows the frequencies (A) and durations (B) of each social behavior studied in both groups. In comparison with the control mice, the PDGFR-β KO mice showed significantly lower frequencies and durations of proximity behaviors (Student's t-test, *p*<0.05; control, n = 9; KO, n = 9). Consistent with these results, the PDGFR-β KO mice showed decreases in the other categories of social behaviors, including social sniffing, active social contact, passive social contact, and mounting (Student's t-test, *p*<0.001; control, n = 9; KO, n = 9), except for duration of social sniffing (Student's t-test, *p*>0.05). On the other hand, the PDGFR-β KO mice showed significantly higher frequencies and durations for approaching and leaving (Student's t-test, *p*<0.001). Videotapes were also analyzed manually afterwards to check aggressive behaviors. However, no aggressive behaviors were observed in both the groups.

### PPI

**Figure 2:**
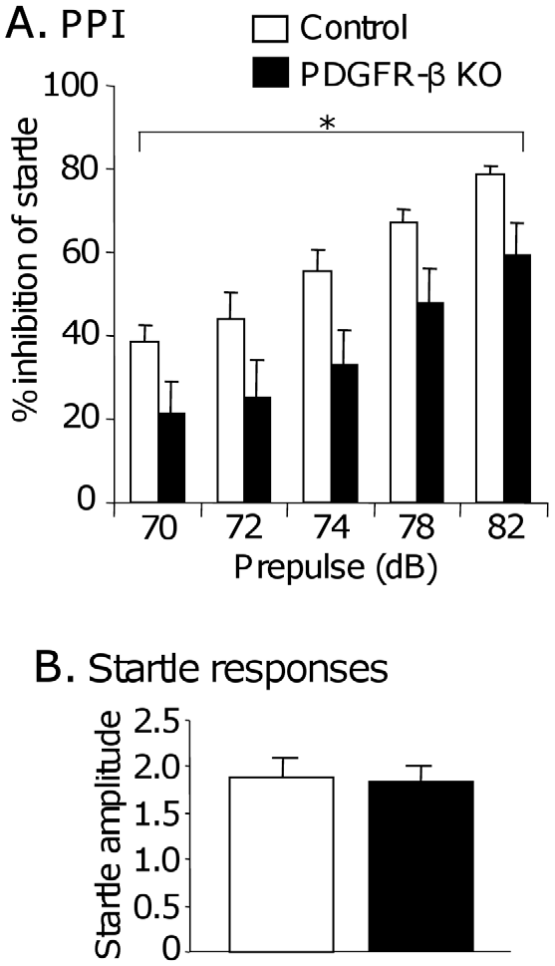
Prepulse inhibition (PPI) of acoustic startle responses. A: PPI (%) at 5 different prepulse intensities (70, 72, 74, 78, and 82 dB). The PDGFR-β KO mice displayed significantly less PPI than the control mice [F(1, 30) = 5.902; *p*<0.05; control, n = 16; KO, n = 16]. B: Acoustic startle amplitudes measured in trials without a prepulse. No significant differences were observed in the acoustic startle amplitudes of the 2 groups of mice. Values indicate the mean ± SE. White columns, control mice, n = 16; black columns, PDGFR-β KO mice, n = 16.

PPI is a paradigm for sensorimotor gating that is most widely used in animal models of schizophrenia and autism. PPI can be easily measured in rodents in a manner almost identical to procedures used in humans [39]–[41]. Statistical comparisons by two-way mixed repeated measures ANOVA indicated that the PDGFR-β KO mice have significant deficits in sensorimotor gating (Figure 2A); there was a significant main effect of group in PPI [F(1, 30) = 5.902; p<0.05; control, n = 16; KO, n = 16], although there was no significant interaction between group and prepulse intensity [F(4, 120) = 0.166; p>0.05; control, n = 16; KO, n = 16]. Furthermore, no significant differences were observed in the amplitudes of the startle responses to the main pulse without a prepulse between the control and PDGFR-β KO mice (Student's t-test, *p*>0.05; control, n = 16; KO, n = 16) (Figure 2B). These results indicate that sensorimotor gating was disturbed in the PDGFR-β KO mice.

### Sensitivity to nociception and fear conditioning

**Figure 3:**
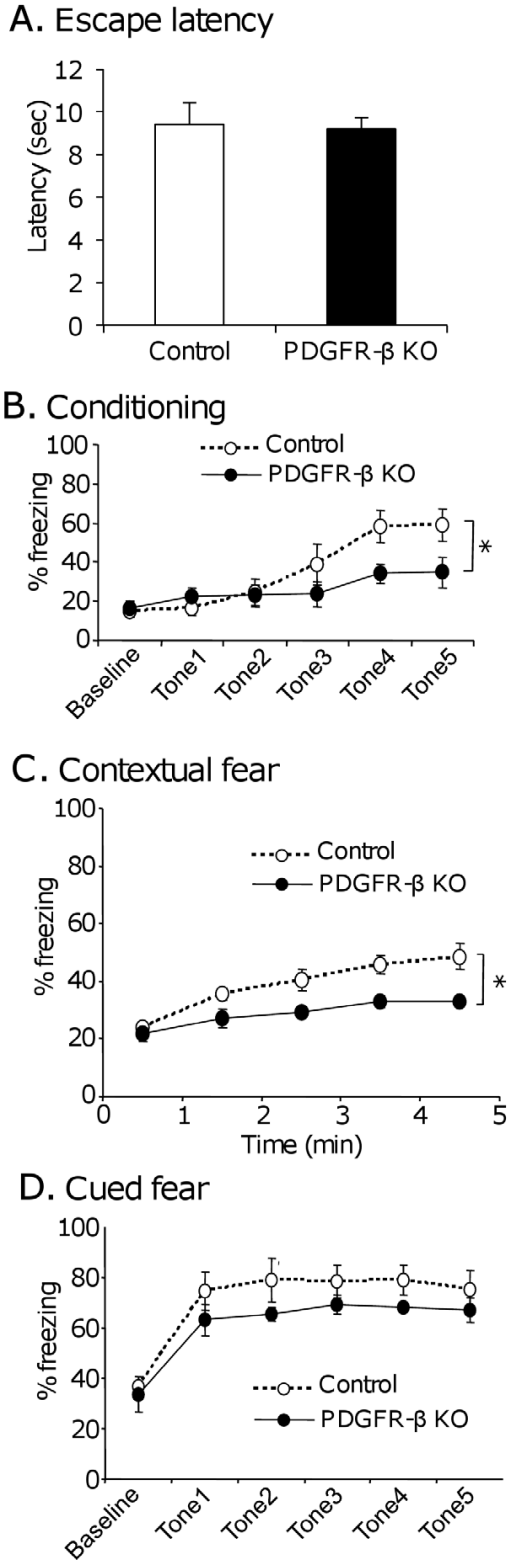
Sensitivity to nociception (A) and freezing behaviors induced by fear conditioning (B–D). A: Mean escape latencies measured in the hot plate test. No significant differences were observed in the mean escape latencies between the control and PDGFR-β KO mice (Student's t-test, *p*>0.05). B: Percent time spent freezing during presentation of each tone associated with an electric shock (tones 1–5). The first shock was presented after tone 1; the mice had not experienced conditioning during repeated presentation of the conditioned tone (tone 1–5). Baseline indicates the percent time spent freezing during the 5 min before tone presentation. The PDGFR-β KO mice spent significantly less time freezing than the control mice in the last 3 conditioning [F(1, 14) = 6.096; *p*<0.05; control, n = 9; KO, n = 7]. C: Percent time spent freezing during the 5-min contextual test. The PDGFR-β KO mice spent significantly less time freezing than the control mice [F(1, 14) = 6.746; *p*<0.05; control, n = 9; KO, n = 7]. D: Percent time spent freezing during the 5 min before tone presentation (baseline) and during presentation of the 20-s conditioned tone (tones 1–5). There were no significant differences in freezing time between the PDGFR-β KO and control mice [F(1, 14) = 2.926; *p*>0.05; control, n = 9; KO, n = 7]. Filled circles, PDGFR-β KO mice; open circles, control mice.

First, sensitivity to nociception was analyzed in the PDGFR-β KO mice. Figure 3A shows the mean escape latencies measured in the hot plate test. Statistical comparisons indicated no significant difference between the control and PDGFR-β KO mice (Student's t-test, *p*>0.05; control, n = 10; KO, n = 9). This finding suggests that sensitivity to nociception in the PDGFR-β KO mice did not differ from that in the control mice.

To test the associative learning capacity of the PDGFR-β KO mice, animals were tested for contextual and cued fear conditioning (Figure 3B–D). In conditioning (Figure 3B), comparison by two-way mixed repeated measures ANOVA (group × conditioning trial No.) indicated that there were marginally significant main effect of group [F(1, 14) = 3.411, p = 0.086; control, n = 9; KO, n = 7], and significant interaction between group and conditioning trial No. [F(4, 56) = 4.173, p = 0.005; control, n = 9; KO, n = 7]. Furthermore, when the data in the last 3 tone-shock conditioning, there was a significant main effect of group [F(1, 14) = 6.096, p<0.05; control, n = 9; KO, n = 7]. These results indicate that the PDGFR-β KO mice had deficits in the ability to associate the cue tone with the electric shock; these deficits were not related to the deficits in nociception.

In the contextual fear retention test (Figure 3C), comparison by two-way mixed repeated measures ANOVA (group × time) indicated that there was a significant main effect of group [F(1, 14) = 6.746, p<0.05; control, n = 9; KO, n = 7], but no significant interaction between group and time [F(4, 56) = 1.367, p>0.05]; control, n = 9; KO, n = 7]. In the cued fear retention test (Figure 3D), comparison by two-way mixed repeated measures ANOVA (group × conditioning trial No.) indicated that there were no significant main effect of group [F(1,14) = 2.926, p>0.05; control, n = 9; KO, n = 7], nor significant interaction between group and conditioning trial No. [F(4, 56) = 0.219, p>0.05; control, n = 9; KO, n = 7]. These findings suggest that learning of tone-shock association and contextual memory were impaired in the PDGFR-β KO mice.

### Forced swimming test

**Figure 4:**
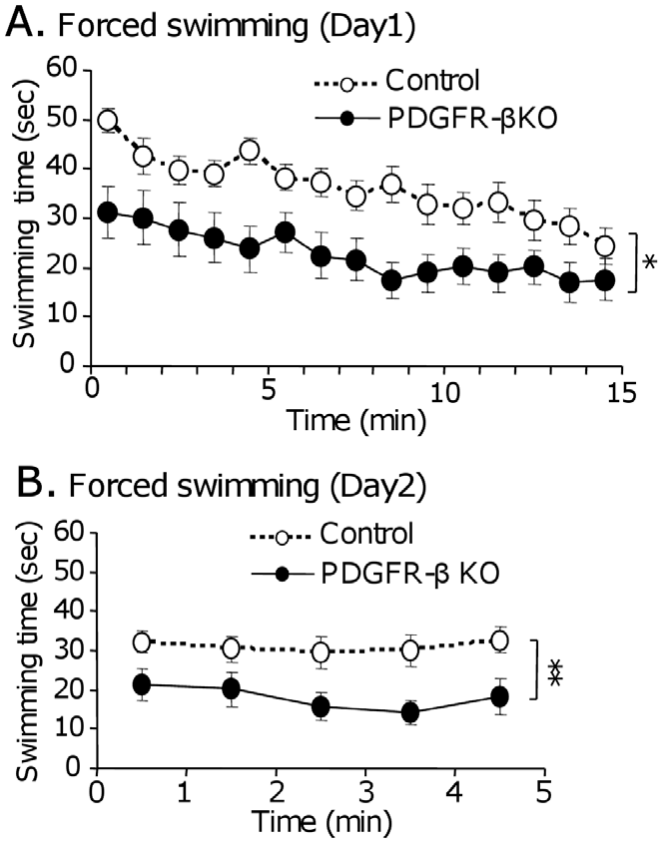
Time course of swimming in the forced swimming test. A–B: Swimming time in 1 min on the first day in the 15-min trial (A) and on the second day in the 5-min trial (B). The PDGFR-β KO mice spent significantly less time swimming than the control mice on the first [F(1, 27) = 11.619; *p*<0.005; control, n = 16; KO, n = 13] (A) and second days [F(1, 27) = 7.235; *p*<0.05; control, n = 16; KO, n = 13] (B). Filled circles, PDGFR-β KO mice; open circles, control mice. *, *p*<0.05; **, *p*<0.005.

The forced swimming test has been previously used to assess depression-related behaviors in animal models that are considered negative symptoms of schizophrenia and autism [42], [43]. In the 15-min test on the first day (Figure 4A), statistical analysis by two-way mixed repeated measures ANOVA (group × time) indicated that there was a significant main effect of group [F(1, 27) = 11.619; p<0.005; control, n = 16; KO, n = 13]. In the 5-min test on the second day (Figure 4B), statistical analysis by two-way mixed repeated measures ANOVA indicated that there was a significant main effect of group [F(1, 27) = 7.235, p<0.05; control, n = 16; KO, n = 13]. These results indicate that the PDGFR-β KO mice became more immobile as the forced swimming test progressed, suggesting that the PDGFR-β KO mice were depressed.

### Food search test

**Figure 5:**
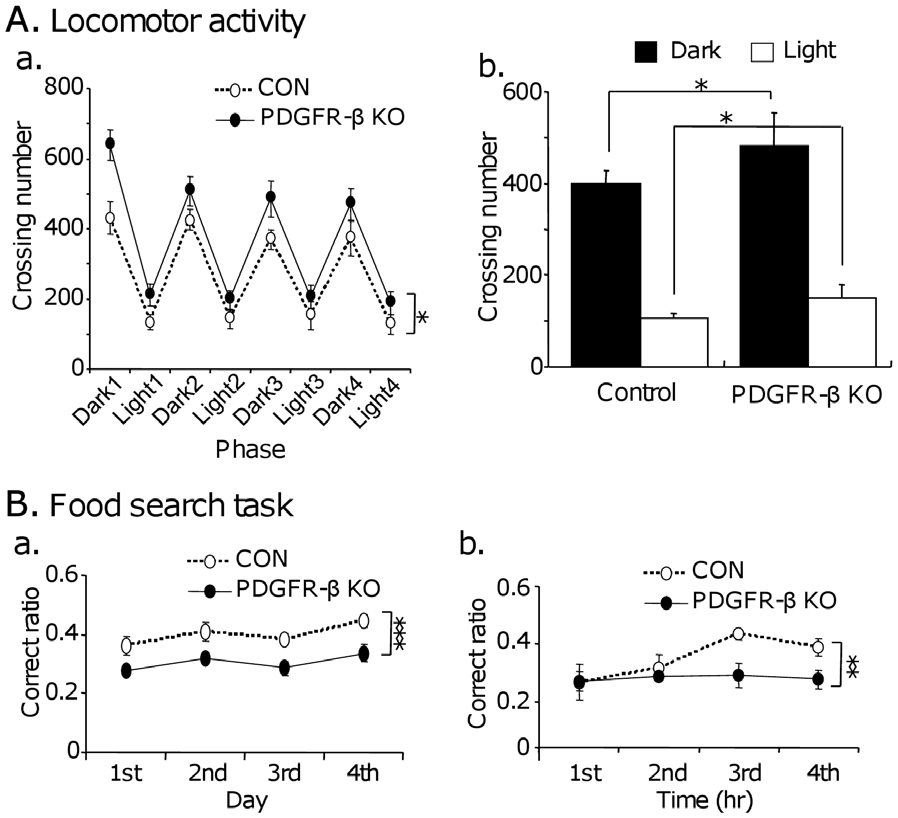
Locomotor activities (A) and spatial performance (B) in the food search test. A: Locomotor activities during the dark and light phases of each experimental day (a) and mean locomotor activities during the dark and light phases of the 4 experimental days (b). The PDGFR-β KO mice displayed significantly higher locomotor activities than the control mice [F(1, 18) = 5.028; *p*<0.05; control, n = 10; KO, n = 10] (a). Mean locomotor activity was also significantly higher in the PDGFR-β KO mice than the control mice during dark and light phases (Student's t-test, *p*<0.05) (b). Vertical axis indicates number of crossing beams. B: Mean correct ratios of each experimental day (a) and each hour after changing the position of the accessible food station (b). The PDGFR-β KO mice displayed significantly higher correct ratios in (a) [F(1, 18) = 16.257; *p*<0.001; control, n = 10; KO, n = 10] across the 4 days and (b) [F(1, 18) = 8.536; *p*<0.01; control, n = 10; KO, n = 10] at 3rd and 4th hr. Filled circles, PDGFR-β KO mice; open circles, control mice. *, *p*<0.05; **, *p*<0.01; ***, *p*<0.001.

The food search test was designed to assess daily activities and spatial memory for food; these activities are particularly sensitive to hippocampal lesions [36]. Figure 5A illustrates locomotor activities during the dark and light phases of each experimental day (a) and mean locomotor activities of the 4 experimental days (b). Statistical analysis by two-way mixed repeated measures ANOVA (group × phase) indicated that there was a significant main effect of group [F(1, 18) = 5.028, p<0.05; control, n = 10; KO, n = 10]. Furthermore, locomotor activities were significantly higher in the PDGFR-β KO mice than the control mice in both dark and light phases (Student's t-test, *p*<0.05).

Figure 5B illustrates the mean correct ratios of each experimental day (a) and each hour after changing the position of the accessible food station (b). Statistical analysis by two-way mixed repeated measures ANOVA (group × day) indicated that there was a significant main effect of group [F(1,18) = 16.257, p<0.001; control, n = 10; KO, n = 10] (Ba). Furthermore, statistical analysis by two-way mixed repeated measures ANOVA (group × time) indicated that there were significant interaction between group and time [F(3,54) = 3.223, p<0.05; control, n = 10; KO, n = 10], and marginally significant main effect of group [F(1,18) = 3.647, p = 0.072; control, n = 10; KO, n = 10] (Bb). When the data at 3rd and 4th hr were analyzed, there was a significant main effect of group [F(1,18) = 8.536, p = 0.01; control, n = 10; KO, n = 10]. These results indicate that spatial learning was significantly disturbed in the PDGFR-β KO mice.

### Histological analyses

**Figure 6:**
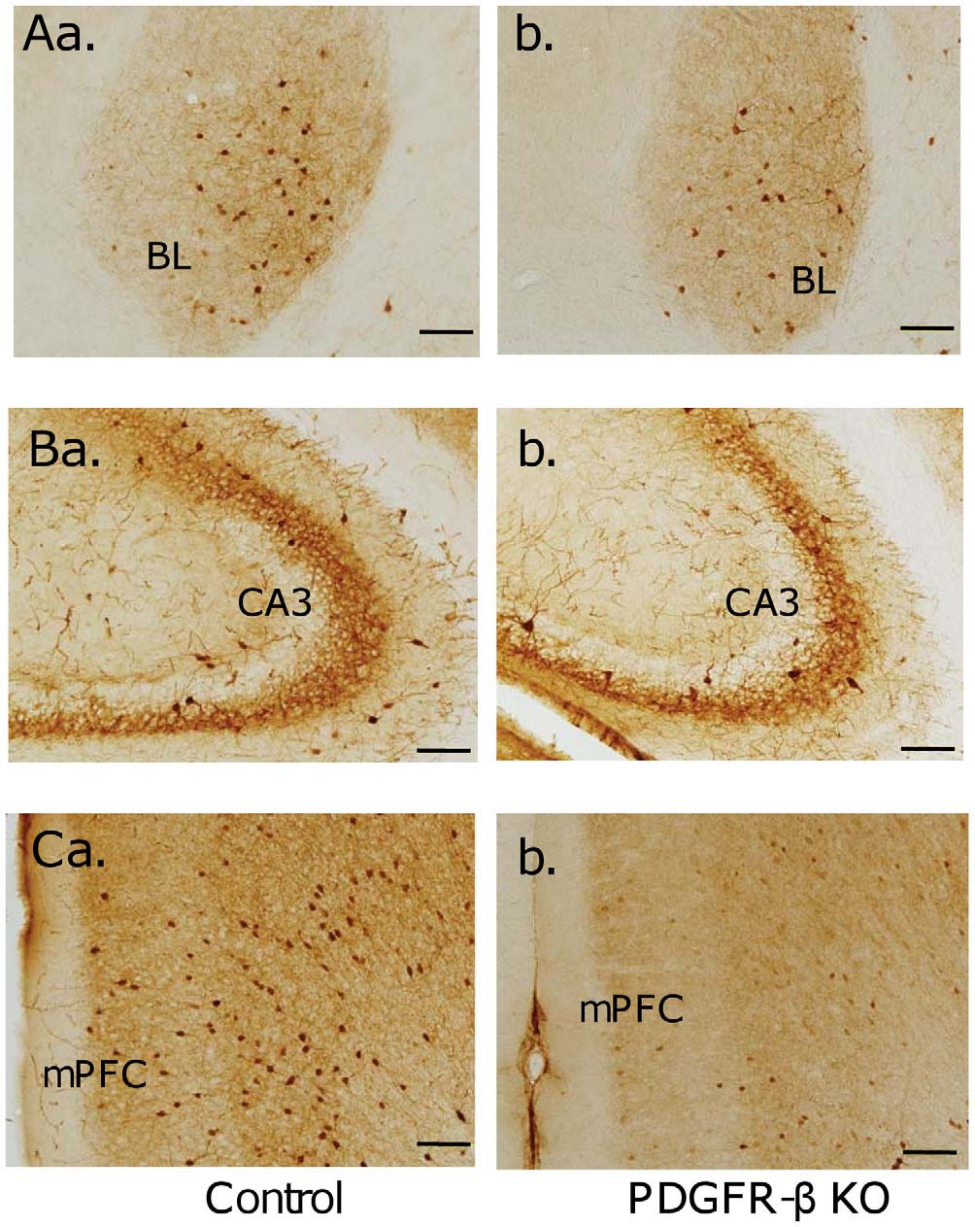
Photomicrographs of the lateral and BL of the amygdala (A), CA3 subfield of the hippocampus (B), and medial prefrontal cortex (C) of the control (a) and PDGFR-β KO (b) mice. Intense labeling of parvalbumin-positive neurons was observed in each area; however, these neurons were less frequently observed in the PDGFR-β KO mice. Scale bar  = 100 µm.

**Figure 7:**
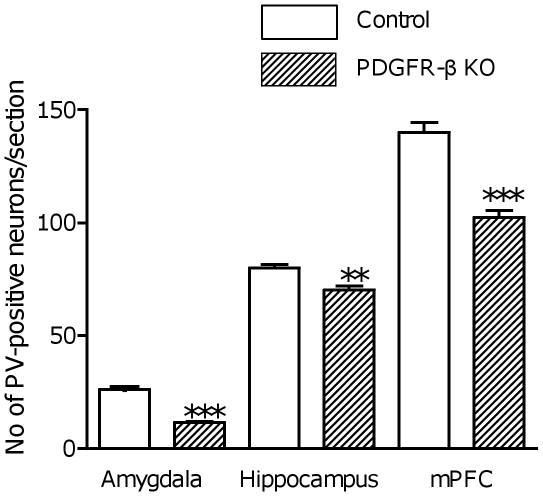
Comparison of the number of parvalbumin-positive neurons in the amygdala, hippocampus, and medial prefrontal cortex between the control and PDGFR-β KO mice. The mean number of parvalbumin-positive neurons was significantly smaller in the amygdala (Student's t-test, *p*<0.001; control, n = 6; KO, n = 6), hippocampus (Student's t-test, *p*<0.005; control, n = 6; KO, n = 6), and medial prefrontal cortex (Student's t-test, *p*<0.001; control, n = 6; KO, n = 6) of the PDGFR-β KO mice compared with the control mice. **, *p*<0.005; **, *p*<0.001.

Previous studies have reported that changes in parvalbumin-expressing GABAergic neurons are important in the pathology of schizophrenia and autism (see Discussion). Figure 6 shows parvalbumin-positive neurons in the lateral and basolateral nuclei of the amygdala (A), CA3 subfield of the hippocampus (B), and medial prefrontal cortex (C) in the control (a) and PDGFR-β KO mice (b). Parvalbumin-positive neurons were less frequently observed in the PDGFR-β KO mice. Figure 7 shows the mean number of parvalbumin-positive neurons in these 3 brain regions (throughout the dorsal hippocampus, amygdala, and medial prefrontal cortex). Statistical analyses indicated that the mean number of parvalbumin-positive neurons was significantly smaller in the amygdala (Student's t-test, *p*<0.001; control, n = 6; KO, n = 6), hippocampus (Student's t-test, *p*<0.005; control, n = 6; KO, n = 6), and medial prefrontal cortex (Student's t-test, *p*<0.001; control, n = 6; KO, n = 6) of the PDGFR-β KO mice compared with the control mice.

### Neurophysiological recordings

**Figure 8:**
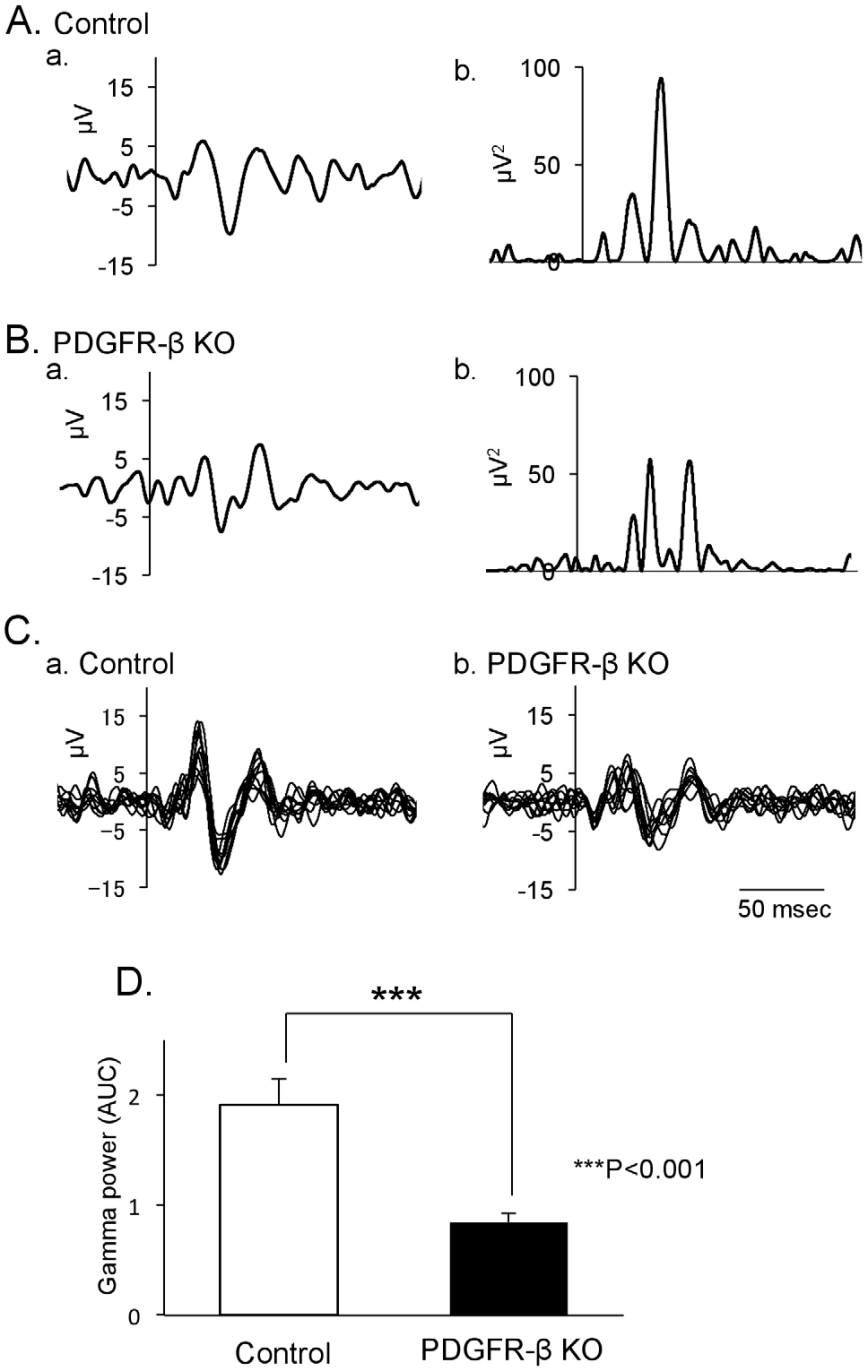
Comparison of evoked event-related gamma power between the control and PDGFR-β KO mice. A–B: Averaged gamma-filtered oscillation (a) and its rectified data (b) recorded from a single control (A) and PDGFR-β KO (B) mice. C: Superimposed illustration of the averaged gamma-filtered oscillation in individual control (a) and PDGFR-β KO (b) mice. D: Comparison of the mean evoked gamma power (AUC) between the control and PDGFR-β KO mice. AUC was significantly low in the PDGFR-β KO mice (Student's t-test, *p*<0.001; control, n = 10; KO, n = 9).

Figure 8 illustrates event-related gamma oscillation in the control and PDGFR-β KO mice. Examples of the averaged gamma-filtered data from individual control (Aa) and PDGFR-β KO mice (Ba) are shown. Superimposed event-related gamma oscillation data of all animals in this study are shown in C (Ca for control mice, n = 10; Cb for PDGFR-β KO mice, n = 9). Gamma oscillation amplitudes were larger in the control mice than the PDGFR-β KO mice. Examples of the rectified gamma data from individual animals are shown in Ab and Bb. Comparisons of the rectified data indicate that the mean evoked gamma power (AUC) was significantly lower in the PDGFR-β KO mice than the control mice (Student's t-test, *p*<0.001) (D).

## Discussion

### Changes in socio-emotional behaviors

In this study, the PDGFR-β KO mice showed decreases in social interactions. Deficits in social interaction are fundamental symptoms of autism, and these deficits are displayed by animal models of autism [43]. Furthermore, this impairment represents the core symptom of schizophrenia (i.e., little interest in social behavior or increased social isolation) [44], [45], and most studies on animal models of schizophrenia describe this impairment as a negative symptom [46]. The PDGFR-β KO mice also showed decreased swimming in the forced swimming test (considered to be a depression-like behavior). This impairment has also been proposed as a negative symptom in animal models of schizophrenia [42]. Similar deficits in the same task have been reported in various animal models of autism [43]. Impairments in GABAergic neurotransmission have been reported to be associated with social disorders and depression-like behaviors [47], [48]. These findings suggest that the behavioral abnormalities observed in the PDGFR-β KO mice might be related to deficits in GABAergic neurotransmission (see below in detail).

### Sensorimotor gating abnormalities

In the present study, the PDGFR-β KO mice displayed deficits in PPI. PPI is an indicator of sensorimotor gating, a process that is important for filtering extraneous sensory information from the external environment. PPI is mediated through a neural system that includes the pontine brainstem, which receives a modulatory input from the nucleus accumbens innervated by the prefrontal cortex, hippocampus, and amygdala [49], and is mediated by various neurotransmitters including GABA in the prefrontal cortex and amygdala [50]–[52]. Deficits in sensorimotor gating have been suggested as a useful endophenotype for the diagnosis of schizophrenia in patients [53] and its respective animal models [54], [55]. Recent data on autism in patients and its respective animal models have indicated PPI deficits [56], [43], [57]. Both of these neuropsychiatric diseases are associated with abnormalities in the fronto-limbic system [56], [58], [27], [59]. In the present study, the number of putative inhibitory parvalbumin-positive neurons was low in the hippocampus, amygdala, and prefrontal cortex. PPI deficits in the PDGFR-β KO mice could be attributed to decreases in GABAergic neurotransmission in the fronto-limbic regions.

### Role of PDGFR-β in learning and memory

In the present study, spatial learning that depends on the hippocampus was disturbed in the PDGFR-β KO mice, as indicated by the results of the food search test. Schizophrenic patients have been reported to show deficits in spatial learning [60], which corresponds to cognitive deficits that are also observed in these patients [61]. Consistently, various animal models of schizophrenia and autism have displayed similar deficits in spatial working memory [61], [43]. Second, emotional learning and retention, which is dependent on the amygdala and hippocampus in cued fear conditioning and contextual memory were also disturbed in the PDGFR-β KO mice. Consistently, human schizophrenic and autistic patients as well as the animal models of these psychiatric disorders show deficits in contextual and cued fear conditioning [62]–[64].

Since previous reports have shown that *PDGFR-β* is expressed in the cortex, hippocampus, amygdala, and brainstem [5], [3], [65], *PDGFR-β* deletion may result in a decrease in the long-term synaptic plasticity of various brain regions. Consistent with this concept, recent studies have reported that PDGF-B is important for long-term potentiation (LTP) in the hippocampus of mice and regulates expression of *Arc/Arg3.1*, a gene that has been implicated in synaptic plasticity and LTP [66], and that LTP is disturbed in the hippocampus of the PDGFR-β KO mice [67]. Furthermore, synaptic plasticity of the amygdala that is induced by Pavlovian fear conditioning is also regulated by the same gene (i.e., *Arc/Arg3.1*) [68]. The present results, along with those of previous studies, strongly suggest that *PDGFR-β* plays an important role in synaptic plasticity relevant to behavioral and cognitive deficits in schizophrenic and autistic patients.

### Role of PDGFR-β in parvalbumin-positive neurons

In the present study, the number of parvalbumin-positive neurons was low in the amygdala and prefrontal cortex including the anterior cingulate cortex of the PDGFR-β KO mice. Since PDGF-B/PDGFR-β signal axis exerts neurotrophic effects on GABAergic neurons [3], [20], a decrease in the number of parvalbumin-positive neurons might be attributed to the loss of neurotrophic effects of PDGFR-β in the PDGFR-β KO mice. Furthermore, PDGFR exerts protective effects on neurons against various brain injuries including oxidative stress [69], [26], [70]. Oxidative stress has been proposed to induce loss and/or maturation impairment in parvalbumin-positive neurons (see review by Behrens and Sejnowski [71]). *PDGFR-β* deletion might increase superoxide, which consequently results in a decrease in parvalbumin-positive neurons in the PDGFR-β KO mice.

Parvalbumin-positive neurons are fast-spiking interneurons [72], [73] that are important for modulating cortical sensory responses [74] and generating gamma oscillations that enhance signal transmission by reducing noise and amplifying signals in cortical circuits [75]. Consistent with these findings, gamma oscillation has been implicated in various cognitive functions, including perception, arousal, attention, learning, and language [76]. Furthermore, parvalbumin-positive GABAergic neurons play a critical role in the development of neural plasticity during the critical period [29]. These results suggest that parvalbumin-positive GABAergic neurons are important for brain development and higher cognitive functions.

Recent studies have reported both changes in gamma oscillation and a reduction in the number of parvalbumin-positive neurons in schizophrenic and autistic patients and respective animal models of these disorders [76], [27], [77], [78], [29], [79]. The present study demonstrated a reduction in evoked gamma oscillation, consistent with studies on human schizophrenic patients [30]–[32]. Evoked gamma oscillation has been linked to sensory registration and top-down cognitive processing (see review by Roach and Mathalon [30]). These findings suggest that socioemotional and cognitive disturbances observed in the PDGFR-β KO mice might be attributed to a reduced number of parvalbumin-positive neurons.

### Conclusions

Although previous reports have indicated that *PDGFR-β* is associated with schizophrenia and autism (see Introduction), no study has reported on the behavioral effects of *PDGF* deletion in mice. The present results demonstrate that the PDGFR-β KO mice share behavioral and neuroanatomical features that are typical of major psychiatric disorders, especially the negative symptoms of schizophrenia, socioemotional disturbances of autism (i.e., abnormalities in social interaction, sensorimotor gating, and cognition), and reduced number of parvalbumin-positive interneurons. Consistently, recent studies have reported that schizophrenia and autism share some genetic and neuroanatomical characteristics, suggesting that the etiological mechanisms of these 2 psychiatric disorders might overlap [80], [81]. Future studies must extensively scrutinize the genetic mechanisms that directly and indirectly affect PDGFR-β functions, and gene encoding PDGFR-β has been implicated as a susceptibility gene for schizophrenic and autistic disorders.
